# Investigating the Spatiotemporal Variability and Driving Factors of Artificial Lighting in the Beijing-Tianjin-Hebei Region Using Remote Sensing Imagery and Socioeconomic Data

**DOI:** 10.3390/ijerph16111950

**Published:** 2019-06-01

**Authors:** Wanchun Leng, Guojin He, Wei Jiang

**Affiliations:** 1 Aerospace Information Research Institute, Chinese Academy of Sciences, Beijing 100094, China; lengwch@radi.ac.cn; 2 University of Chinese Academy of Sciences, Beijing 100049, China; 3 Key Laboratory of Earth Observation Hainan Province, Sanya 572029, China; 4 China Institute of Water Resources and Hydropower Research (IWHR), Beijing 100038, China

**Keywords:** artificial lighting, DMSP/OLS, spatio-temporal patterns, driving factors, socioeconomic data, nighttime light pollution, Beijing-Tianjin-Hebei region, remote sensing

## Abstract

With rapid urbanization and economic development, artificial lighting at night brings convenience to human life but also causes a considerable urban environmental pollution issue. This study employed the Mann-Kendall non-parametric test, nighttime light indices, and the standard deviation method to investigate the spatio-temporal characteristics of artificial lighting in the Beijing-Tianjin-Hebei region. Moreover, nighttime light imagery from the Defense Meteorological Satellite Program Operational Linescan System, socioeconomic data, and high-resolution satellite images were combined to comprehensively explore the driving factors of urban artificial lighting change. The results showed the following: (1) Overall, there was an increasing trend in artificial lighting in the Beijing-Tianjin-Hebei region, which accounted for approximately 56.87% of the total study area. (2) The change in artificial lighting in the entire area was relatively stable. The artificial lighting in the northwest area changed faster than that in the southeast area, and the areas where artificial lighting changed the most were Beijing, Tianjin and Tangshan. (3) The fastest growth of artificial lighting was in Chengde and Zhangjiakou, where the rates of increase were 334% and 251%, respectively. The spatial heterogeneity of artificial lighting in economically developed cities was higher than that in economically underdeveloped cities such as Chengde and Zhangjiakou. (4) Multi-source data were combined to analyse the driving factors of urban artificial lighting in the entire area. The Average Population of Districts under City (**R*^2^* = 0.77) had the strongest effect on artificial lighting. Total Passenger Traffic (**R*^2^* = 0.54) had the most non-obvious effect. At different city levels, driving factors varied with differences of economy, geographical location, and the industrial structures of cities. Urban expansion, transportation hubs, and industries were the major reasons for the significant change in nighttime light. Urban artificial lighting represents a trend of overuse closely related to nighttime light pollution. This study of artificial lighting contributes to the rational planning of urban lighting systems, the prevention and control of nighttime light pollution, and the creation of liveable and ecologically green cities.

## 1. Introduction

With the rapid development of urbanization and economies, urban artificial lighting has greatly developed [1]. Charming city night scenes have provided citizens with safer, more comfortable and convenient lives. It has also reduced the occurrence of traffic accidents and crimes, and artificial lighting is considered a symbol of a city’s civilization [2]. China has the world’s highest rate of urban expansion [3]. As one of the most important economic and urbanization growth regions in China, the Beijing-Tianjin-Hebei region has absorbed large inflows of migrants in recent years, providing much labour input and bringing growth in artificial lighting facilities. As an important representation of the expansion of human activities, artificial lighting poses adverse influences on ecological environments [4], human health [5], and ecosystem stability [6,7]. In recent years, the unreasonable use of artificial lighting facilities has shown an explosive increasing trend; nighttime light pollution has gradually become a potential environmental problem and is a widely discussed and studied topic around the world [8,9]. However, urban artificial lighting has not yet been integrated into unified planning in China [10], and there is a lack of attention to urban artificial lighting environment issues. Therefore, urgent demands have been placed on the study and rational planning of artificial lighting.

The investigation of artificial lighting using ground surveys makes it difficult to explore the characteristics of artificial lighting at larger scales [11]. Nighttime light remote sensing technology has the advantages of being large-scale, objective, and of low cost, and it can accurately capture the nighttime light information of human activity regions [12]. Defense Meteorological Satellite Program Operational Linescan System (DMSP/OLS) has provided the world’s longest sequence of nighttime light remote sensing imagery from 1992 to 2013, which has had extensive application in previous studies [13,14,15,16,17,18]. In the regional spatiotemporal variability analysis of nighttime lights by using time-series imagery, Bennie et al. used the mean-average method to analyse the trends of artificial lighting pollution in Europe for the past 15 years and pointed out the areas where nighttime light pollution had sharply increased or decreased [19]. Han et al. found that the growth areas of China’s artificial lighting were mainly located in large coastal cities in the east [20]. However, these studies have focused on the analysis of nighttime light changes using different time-series of imagery, and there remains a lack of integration of the changing trends and fluctuation characteristics of urban nighttime lights to analyse spatiotemporal variability. Moreover, studies have shown that the fast-developing urban circles have experienced increased nighttime light pollution in the past few decades [21]. Chalkias et al. used time-series DMSP/OLS nighttime light images and Geographical Information System techniques to find that urban suburbs were suffering serious light pollution due to the rapid development of urbanization [4]. Helga U. Kuechly used high-resolution nighttime light images to monitor the impacts of different types of land use in Berlin on artificial lighting, which showed that street lighting was the main source of artificial lighting in this region [9]. Ma proposed a spatially explicit approach for different scales of nighttime light research and found that the spatial expansions of artificial nighttime light brightness geographically correspond to urban expansion gradients [22]. However, the quantitative monitoring and measurement of nighttime lights has been rarely considered. In addition, research on the correlations between nighttime light and socioeconomical indices have extensive applications. However, most of them have been applied to analyse socioeconomic phenomena, such as urban expansion [14], economic development [23], energy consumption [24], population density [25] and transportation mobility [26], there currently remains a lack of documents to comprehensively reveal the driving factors of artificial lighting change.

This study intends to explore the long-term spatiotemporal variability and causes of the artificial lighting changes in the Beijing-Tianjin-Hebei region by combining multi-source remote sensing imagery and socioeconomic data. Methodologies for combining changing trends and fluctuation characteristics are proposed to investigate the regional spatiotemporal trends of artificial lighting. Then, the study employs quantitative indicators to measure the nighttime lights at city levels. In addition, socioeconomic data and high-resolution satellite imagery are coupled to explore the driving factors from the perspective of socioeconomic indices and city levels. These findings are of great significance for the rational use of artificial lighting and the control of nighttime light pollution. In addition, the methodologies of this study have good applicability to research on the spatiotemporal variability of nighttime lights and the exploration of driving factors, and the research framework of this study can also be used for nighttime lights research and light pollution prevention and control in other regions of the world.

## 2. Study Area and Data Sources 

### 2.1. Study Area

**Figure 1:**
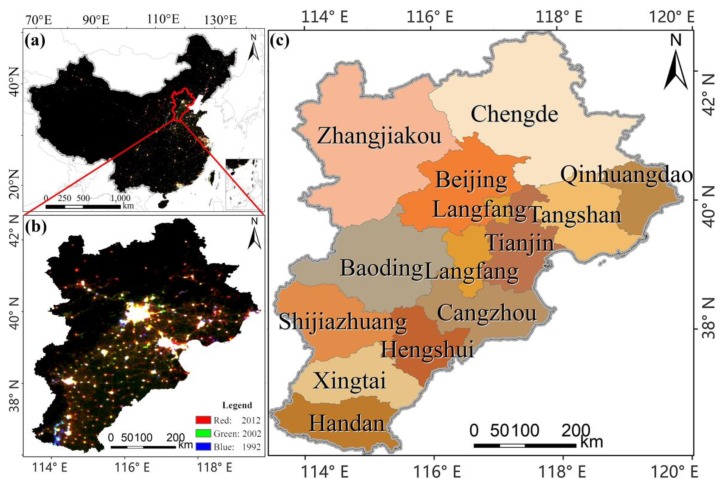
Study area (colour composite of nighttime lights of 1992 (blue), 2002 (green), 2012 (red)): (**a**) colour composite of China; (**b**) colour composite of the study area, the Beijing-Tianjin-Hebei region; (**c**) administrative division map of the study area.

The Beijing-Tianjin-Hebei region is the third largest economic growth region in China and includes Beijing, Tianjin and 11 prefecture-level cities in Hebei Province. The region occupies 2% of the country’s total area [27] and has a permanent population of 110 million. According to the National Bureau of Statistics of China, the GDP of the Beijing-Tianjin-Hebei region in 2016 reached 68,857.15 billion-yuan, which accounts for 9.25% of the national total. The rapid development of this area has attracted more population and enterprises, bringing about economic development and inevitably leading to the overuse of artificial lighting. The investigation of the characteristics and driving factors of artificial lighting in this area can contribute to the rational use and control of artificial lighting in the future and is of great significance to the realization of intelligent lighting systems and smart city systems. It is an important requirement for the integration of Beijing-Tianjin-Hebei region development. Figure 1 shows the study area. 

### 2.2. Data Sources and Processing

#### 2.2.1. DMSP/OLS Nighttime Light Imagery

DMSP/OLS provides a new data acquisition method compared with traditional optical sensors. It can operate at night, which enables the monitoring of urban artificial lighting and low-intensity light emitted by small-scale residential and vehicular traffic [11], and thus provides the basis for extensive research and application of nighttime light remote sensing. This study uses version 4 DMSP/OLS stable nighttime light data downloaded from NOAA’s National Centres for Environmental Information (NCEI). The stable nighttime light imagery composites are for one period of a year, and the effects of background noises, such as wildlife fire, moonlight, and clouds, have been removed [28]. The range of data digital numbers (DN) is 0–63. We selected 33 period images as the study dataset, acquired by six different sensors from 1992 to 2012. 

The DMSP/OLS has no on-board calibration for the visible band. Because the nighttime light images span a long time and are acquired by six different sensors (F10, F12, F14, F15, F16, and F18), it is necessary to correct the images to eliminate the influence of time and sensor differences. The image correction includes intercalibration, annual composition, and annual series correction. 

*Intercalibration*: Intercalibration can eliminate the influence of sensor differences and was established using the invariant area method proposed by Elvidge et al. [29]. The correction model is given as follows:

(1) $$DN_{cal}=C_{0}+C_{1}\times DN_{n}+C_{2}\times DN_{n}^{2}$$

where *DN_cal_* represents the pixel value of the calibrated image and *DN_n_* represents the pixel value of the original image in year *n*. *C*_0_, *C*_1_, and *C*_2_ are the coefficients of the regression model, which can be obtained following the empirical procedure [29]. 

*Annual composition*: There may be multiple nighttime light images of different sensors in one year. Based on the intercalibration, the annual composition is employed to synthesize multiple images into one image a year. The formula is as follows:

(2) $$DN_{\left(i,n\right)}=\left\{ \begin{array}{l} \;0,\;DN_{\left(i,n\right)}^{a}=0\;and\;DN_{\left(i,n\right)}^{b}=0\\ \left(DN_{\left(i,n\right)}^{a}+DN_{\left(i,n\right)}^{b}\right)/2,\;other \end{array}\right.$$

where $DN_{\left(i,n\right)}^{a}$ and $DN_{\left(i,n\right)}^{b}$ represent the *i*th pixel values of sensor *a* and sensor *b* in year *n* after the intercalibration, respectively. $DN_{\left(i,n\right)}$ represents the *i*th pixel value in year *n* after the annual composition.

*Annual series correction*: The annual series correction can eliminate the influence of long times. According to the trend of urban expansion [30], the principle that assumes the DN values in the latter year are no less than those in the previous year in the same position is determined [18], and the correction formula is given as follows:

(3) $$DN_{\left(i,n\right)}=\left\{ \begin{array}{l} \;0,\;DN_{\left(i,n+1\right)}=0\;\\ DN_{\left(i,n-1\right)},\;DN_{\left(i,n+1\right)}>0\;and\;DN_{\left(i,n-1\right)}<0\\ \;DN_{\left(i,n\right)},\;other \end{array}\right.$$

where *DN*_(*i*,*n*−1)_, *DN*_(*i*,*n*)_, *DN*_(*i*,*n*+1)_ represent the *i*th pixel values in year *n*−1, *n*, and *n*+1, respectively, after annual composition.

*Image post processing*: The corrected images are clipped, resampled and reprojected to obtain the images of the study area, which are then converted into the WGS_1984 projection with the WGS_1984 ellipsoid.

#### 2.2.2. Socioeconomic Data

Most studies have shown that socioeconomic parameters, such as population [25,31,32], economy [33,34], transportation [26], and electricity consumption [23,24], are related to nighttime lighting. This study couples the socioeconomic data and nighttime light images to explore the driving factors of urban artificial lighting change. Based on the studies of Shi et al. [23] and Jiang et al. [35], eight light-related socioeconomic parameters are selected from the *China City Statistical Yearbook*, which contains statistical data on the population, economy, transportation and electricity consumption from 1992 to 2012 of the 13 prefecture-level cities in the Beijing-Tianjin-Hebei region. The population parameters include Annual Average Population of Total City (AAPTC) and Annual Average Population of Districts under City (AAPDC). The economy parameters include Gross Regional Product of Total City (GRPTC) and Gross Regional Product of Districts under City (GRPDC). The transportation parameter includes Total Passenger Traffic (TPT). The electricity parameters include Annual Electricity Consumption (AEC), Electricity Consumption for Industrial (ECI), and Household Electricity Consumption for Urban and Rural Residential (HECURR).

#### 2.2.3. High-Resolution Satellite Remote Sensing Imagery

High-resolution satellite images of some typical areas where nighttime lights change sharply are obtained from Google Earth (https://earth.google.com). The study aims to explore the driving factors of artificial lighting change at the local scale by monitoring the land cover changes in these typical areas. The high-resolution images on Google Earth are mainly from DigitalGlobe and the sensors include IKONOS, Pléiades-1, QuickBird, WorldView-2, GeoEye-1, and KOMPSAT-2. The resolution of these images is better than 1 metre, which can clearly reflect the land cover change, and the parameters of these high-resolution satellite images are shown in Table 1.

## 3. Methods

**Figure 2:**
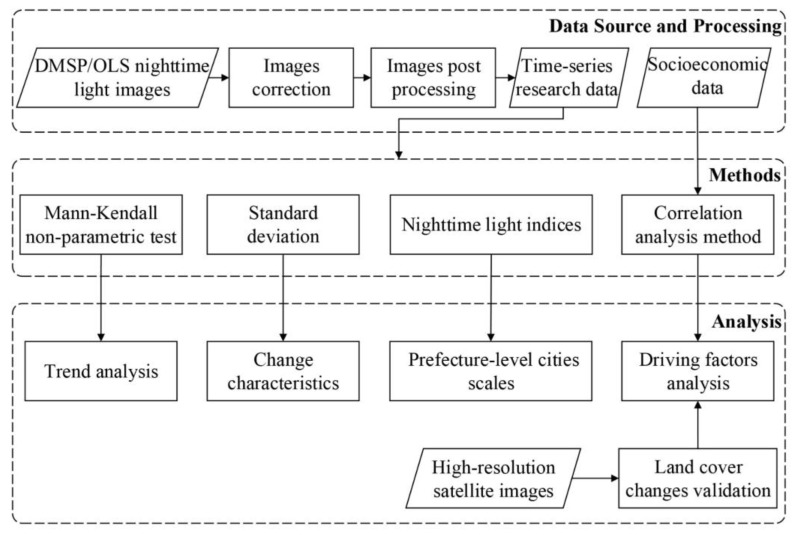
Flowchart of methodology for investigation of the characteristics and driving factors of artificial lighting in the Beijing-Tianjin-Hebei region.

Figure 2 shows the flowchart of the methodology for investigating artificial lighting in the Beijing-Tianjin-Hebei region.

### 3.1. Trend Analysis

#### 3.1.1. Mann-Kendall Non-Parametric Test Method (M-K Test)

The M-K test is calculated to reveal the spatio-temporal fluctuation characteristics of artificial lighting changes. The M-K test is a non-parametric trend analysis method that has the advantages of being unaffected by the actual distribution of samples and can evade the problems caused by outliers [36]. Currently, it is widely used to reflect change trends in long-term sequences [37]. 

The M-K test statistic is:

(4) $$U=\frac{\tau }{\sqrt{Var\left(\tau \right)}}$$

where:

(5) $$\tau =\frac{4S}{n\left(n-1\right)-1}$$

(6) $$S=\sum_{i=1}^{n}\sum_{j=i+1}^{n}\mathrm{sgn}\left(x_{j}-x_{i}\right)$$

(7) $$\mathrm{sgn}\left(x_{j}-x_{i}\right)=\left\{ \begin{array}{l} 1,x_{j}>x_{i}\\ 0,x_{j}=x_{i}\\ -1,x_{j}<x_{i} \end{array}\right.$$

(8) $$Var\left(\tau \right)=\frac{2\left(2n+5\right)}{9n\left(n-1\right)}$$

The time series is $x_{i}$ (*i* = 1, 2, …, n); *i* = 1, 2, …, *n*; *j* = *i* + 1, *i* +2, …, *i* + *n*. The range of *U* values is (−11.99, 12.35), which is stretched along the colour ramp in ArcGIS 10.3 (Esri). If *U* > 0, the artificial lighting is increasing, and the higher the value, the more significant the increase. Conversely, if *U* < 0, the artificial lighting is decreasing, and the lower the value, the more significant the decrease.

#### 3.1.2. Standard Deviation

The standard deviation represents the average value of the data deviation from the normal distance and the degree of dispersion of the time-series data. This study uses the standard deviation to reflect the fluctuation characteristics of artificial lighting; the higher the value, the greater the interannual change of artificial lighting [38]. The calculation formula is as follows:

(9) $$Stdev=\sqrt{\frac{1}{n}\sum_{i=1}^{n}{\left(DN_{i}-\bar{DN}\right)}^{2}}$$

where *Stdev* is the standard deviation. In ArcGIS 10.3, the Natural Breaks (Jenks) method is used to reclassify the standard deviation into five categories: highest (*Stdev* > 11.93), high (6.82 < *Stdev* <11.93), medium (3.30 < *Stdev* < 6.82), low (0.96 < *Stdev* ≤ 3.30), and lowest (0 < *Stdev* ≤ 0.96).

### 3.2. Construction of the Nighttime Light Indices

This study constructs three kinds of nighttime light indices (total night light (TNL), mean night light (MNL), and standard deviation of night light (SDNL) [18]) to analyse the spatio-temporal characteristics of artificial lighting changes in the Beijing-Tianjin-Hebei region:

(1) TNL refers to the total amount level of artificial lighting in the study area. The formula for it is:

(10) $$TNL=\sum_{i=1}^{63}C_{i}DN_{i}$$

where *DN_i_* is the *i*th pixel value and C*_i_* is the number of pixels that corresponds to the pixel value.

(2) MNL refers to the average level of artificial lighting in the study area. The formula for it is:

(11) $$MNL=\left(\sum_{i=1}^{63}C_{i}\times DN_{i}\right)/\sum_{i=1}^{63}C_{i}$$

(3) SDNL refers to the spatial heterogeneity level of artificial lighting in the study area. The formula for it is:

(12) $$SDNL=\sqrt{\frac{1}{N-1}\sum_{i=1}^{N}{\left(DN_{i}-MNL\right)}^{2}}$$

where *N* is the total number of pixels in the study area.

### 3.3. Correlation Analysis Method

The linear fitting regression method and the exponential fitting regression method are used to analyse the correlation of different socioeconomic factors and TNL, which can be used to analyse the driving factors of urban artificial lighting pollution. **R*^2^* is used to determine the correlation of the fitting results; the larger the value, the stronger the correlation. *T*-test and *F*-test, both as significance test, are used to verify the significance of the fitting models and value of *p* is used to measure the significance. When the value is less than 0.05 (*p* < 0.05), the fitting result is significant, and when the value is less than 0.01 (*p* < 0.01), the fitting result is strongly significant with statistical significance [33,34].

## 4. Results

### 4.1. Analysis of the Characteristics of Artificial Lighting in the Beijing-Tianjin-Hebei Region

#### 4.1.1. The Trend Analysis of Artificial Lighting

**Figure 3:**
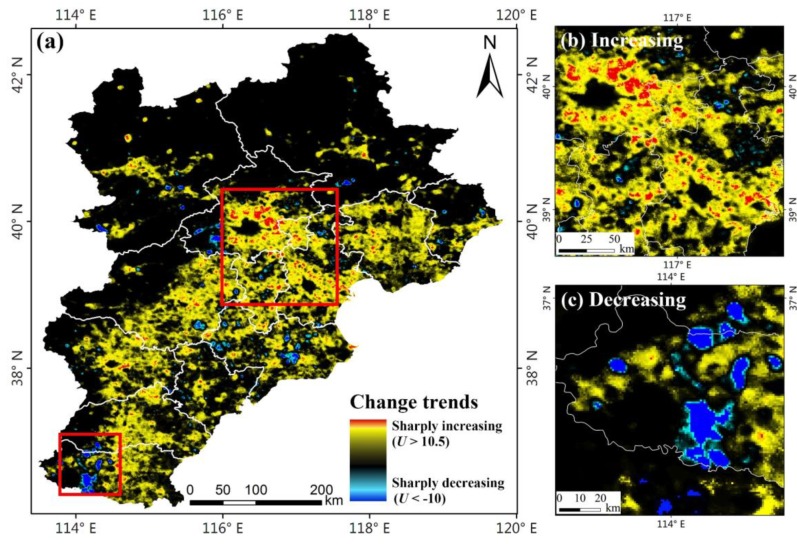
(**a**) Change trend of artificial lighting in the Beijing-Tianjin-Hebei region from 1992 to 2012; (**b**) main areas with the most serious artificial lighting increase; (**c**) main areas with artificial lighting decrease.

The Mann-Kendall non-parametric test method was used to analyse the spatio-temporal characteristics of artificial lighting in the Beijing-Tianjin-Hebei region, as shown in Figure 3. The red-yellow region (0 < *U* ≤ 12.35) represents the intensification area of artificial lighting, and the red region (10.5 < *U* ≤ 12.35) represents that the artificial light has sharply increased. The dark blue-light blue region (−11.99 ≤ *U* < 0) represents the reduction area of artificial lighting, and the dark blue region (−11.99 ≤ *U* < −10) represents that the artificial light has sharply decreased. The black region (*U* = 0) represents no change of the artificial lighting or the non-lighting area.

Figure 3a shows that the artificial lighting in the study area had an overall increasing trend from 1992 to 2012 and that the growth area accounted for 56.87% of the entire study area. The growth of artificial lighting was the most serious in Beijing and Tianjin, as shown in Figure 3b, followed by these two cities as the core extended outward; the growth of artificial lighting in Langfang City, Tangshan City, and east of Baoding City was also obvious. There were few areas with reduced artificial lighting, mainly distributed in Handan City, as shown in Figure 3c, followed by Cangzhou City and then some suburbs of Beijing City and Zhangjiakou City. There were two types of areas where the artificial lighting had no change. One was the core urban area of the city, where the nighttime lights on the images were saturated (DN = 63), mainly in Beijing, Tianjin, and Shijiazhuang City. The other was a non-nighttime light area, mainly in suburban areas and non-residential areas. There was basically no artificial lighting in this area, which was mainly distributed in the northwest of Chengde City and the north of Zhangjiakou City.

#### 4.1.2. Change Characteristics Analysis of Artificial Lighting

**Figure 4:**
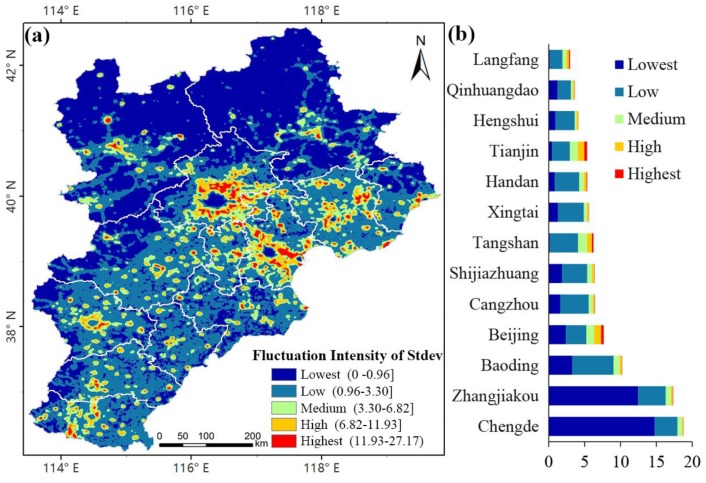
Standard deviation of artificial lighting changes in the Beijing-Tianjin-Hebei region from 1992 to 2012: (**a**) fluctuation characteristics of Stdev; (**b**) proportion of Stdev at prefecture-level cities (%).

Based on the time-series nighttime light data from 1992 to 2012, the standard deviations of time-series images were calculated to analysis the change characteristics of artificial lighting. Figure 4a shows that the overall trend of artificial lighting changes was stable from 1992 to 2012; most of the study area had low fluctuations and only a small part of the study area fluctuated greatly. The fluctuation of the northwest study area was low and that of the southeast was high. In the spatial distribution, high-fluctuation regions (red-orange) and medium-fluctuation regions (light-green) were mainly concentrated in the peripheral expansion areas of core urban regions and the coastal areas of the ports; these areas accounted for a small proportion, 6.64% and 9.08%, respectively. Low-fluctuating regions accounted for a large proportion (84.3%) and were mainly distributed in suburban areas, non-residential areas, and urban core areas. There was either no artificial lighting or a slow increase in lighting in these areas.

Figure 4b shows the proportion of different levels of artificial lighting in different prefecture-level cities, among which Beijing, Tianjin, and Tangshan City had the largest proportion of high-fluctuation and medium-fluctuation regions, and artificial lighting increased obviously. Langfang, Shijiazhuang, and Baoding also accounted for a large proportion of high-fluctuation and medium-fluctuation regions; especially in the northeast of Langfang, caused by the population and industrial expansion in Beijing and Tianjin. In addition, Tangshan City had the lowest proportion of low-fluctuation regions, followed by Langfang and Tianjin City. 

### 4.2. Analysis of Artificial Lighting at Prefecture-Level Cities in the Beijing-Tianjin-Hebei Region

**Figure 5:**
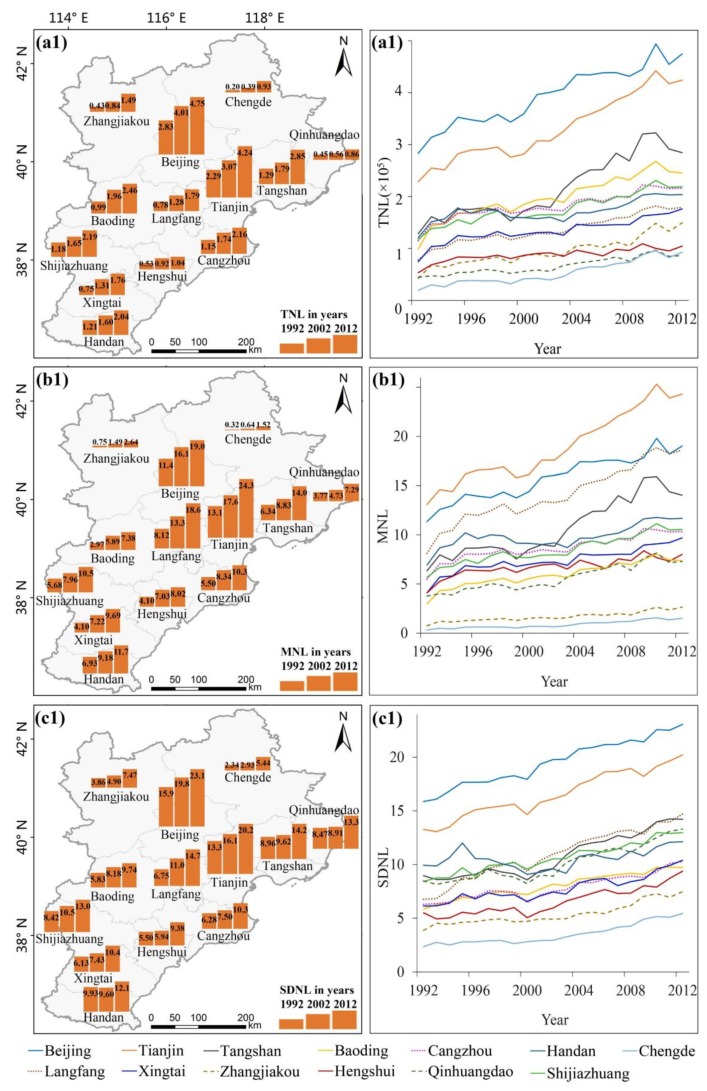
(**a1**,**a2**) TNL and its change trend of cities at the prefecture level in the Beijing-Tianjin-Hebei region; (**b1**,**b2**) MNL and its change trend of cities at the prefecture level in the Beijing-Tianjin-Hebei region; (**c1**,**c2**) SDNL and its change trend of cities at the prefecture level in the Beijing-Tianjin-Hebei region.

Three indicators, TNL, MNL, and SDNL, were selected to analyse the artificial lighting characteristics of 13 prefecture-level cities in the Beijing-Tianjin-Hebei region. Figure 5(a1) reflects the distribution of TNL at different prefecture-level cities, and the histograms represent the TNL of 1992, 2002 and 2012. The figure clearly shows that the TNL in Beijing, Tianjin and Tangshan was the highest and that in Chengde, Qinhuangdao, and Hengshui was the lowest. TNL was not only related to the level of artificial lighting but was also related to the urban area. Some cities with high growth of artificial lighting but small area also had low TNL, such as Langfang City. Figure 5(a2) reflects that TNL in the Beijing-Tianjin-Hebei region showed an overall increasing trend, of which Chengde City had the fastest growth rate (374%), followed by Zhangjiakou (251%). The growth rates of Beijing and Tianjin were 68% and 86%, respectively. Overall, TNL increased rapidly in the economically underdevelopment regions but slowly in the economically developed regions.

Compared with TNL, MNL can eliminate the influence of urban area and reflect the spatial density of artificial lighting in the study area. Figure 5(b1) reflects the distribution of MNL in different cities, and the histograms represent the MNL of 1992, 2002 and 2012. MNL in economically developed cities like Tianjin, Beijing, and Langfang was the highest, and that in economically underdeveloped cities like Chengde, Zhangjiakou and Qinhuangdao was the lowest. The Beijing-Tianjin-Langfang triangle economic development zone centred on Beijing had a rapid economic development. The MNL was approximately 20 (DN = 20) in these areas, and the artificial lighting pollution was the most serious. Figure 5(b2) reflects that MNL in the Beijing-Tianjin-Hebei region showed an overall growth trend the same as that of TNL. Among them, the growth rate of MNL in Chengde was the fastest, with a growth rate of 374%. Moreover, the MNL was not affected by area of cities and was high in Langfang City, where as the TNL of that was low.

SDNL was used to reflect the spatial heterogeneity of artificial lighting in different areas. Figure 5(c1) reflects the distribution of SDNL in different cities; the histograms represent the SDNL of 1992, 2002 and 2012. In Beijing, Tianjin, Langfang and other economically developed regions, SDNL was great, whereas in Chengde, Zhangjiakou and other economically underdeveloped regions SDNL was relatively small. Figure 5(c2) shows that the SDNL of artificial lighting in the study area from 1992 to 2012 had an overall growth trend. The SDNL in Chengde grew the fastest (132% growth rate), which was the result of rapid economic growth, followed by Langfang (118%). Figure 3 shows that the growth of artificial lighting in Langfang City was uneven and there was a large spatial heterogeneity; the artificial lighting in region near Beijing and Tianjin grew faster, whereas the region far from Beijing and Tianjin grew slowly.

### 4.3. Analysis of Driving Factors of Artificial Lighting in the Beijing-Tianjin-Hebei Region

**Figure 6:**
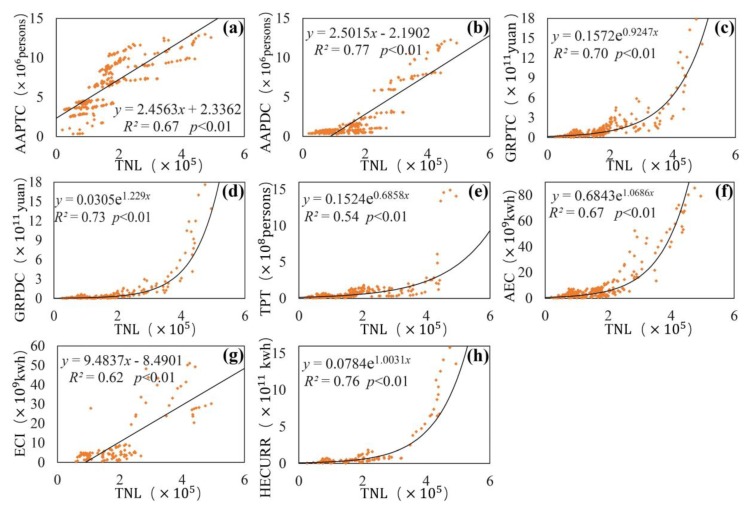
Regression analysis of different socioeconomic parameters and TNL. Population parameters: (**a**) AAPTC, (**b**) AAPDC; Economic parameters: (**c**) GRPTC, (**d**) GRPDC; Traffic parameters: (**e**) TPT; Energy parameters: (**f**) AEC, (**g**) ECI, (**h**) HECURR.

This study selected eight socioeconomic parameters of population, economy, traffic, and energy to analyse the driving factors of artificial lighting. The regression analysis results of different socioeconomic parameters and TNL are shown in Figure 6. The horizontal axis represents the TNL of 13 cities from 1992 to 2012, and the vertical axis corresponds to the socioeconomic parameters of 13 cities from 1992 to 2012. 

The population parameters include AAPTC and AAPDC. Figure 6a,b are the linear regression results of the two indicators and TNL. The *R*^2^ (0.67) of AAPTC and the *R*^2^ (0.77) of AAPDC were high, and both passed the significance test of 0.01. The result shows that the population parameters had a certain impact on the artificial lighting and that the AAPDC had a strong influence on light. Although the correlation between population parameters and TNL was strong for the entire study area, there were also obvious differences between different cities, which resulted in a relatively large degree of dispersion of sample points. 

Figure 6c,d are the regression results of the two economic parameters (GRPTC and GRPDC) and TNL. Both parameters had perfect exponential fitting with TNL, and the *R*^2^ (0.70) of GRPTC and the *R*^2^ (0.73) of GRPDC were both larger than 0.7, which showed strong correlations. 

Traffic parameters can reflect the activity of the population and the economy. Figure 6e shows the regression analysis results of TPT and TNL. The *R*^2^ (0.54) of TPT passed the significance test of 0.01. However, compared with the other parameters, passenger traffic had the smallest impact on artificial lighting and the weakest correlation. Transportation lighting plays an important role in artificial lighting, however, there was a large difference of TPT in the different cities in Figure 6e; for example, the TPT in Beijing far exceeded that in other economically underdeveloped cities. 

Figure 6f–h are the results of the regression analysis of the three electricity energy parameters and TNL, all of which passed the significance test of 0.01. Among them, the *R*^2^ (0.76) of HECURR was the highest, with the strongest correlation with artificial lighting. HECURR mainly included nighttime light in residential areas, which was the main source of artificial lighting. AEC also had a strong correlation with artificial lighting, although AEC differences were relatively large in different cities. The correlation between ECI and artificial lighting was relatively weak. 

**Figure 7:**
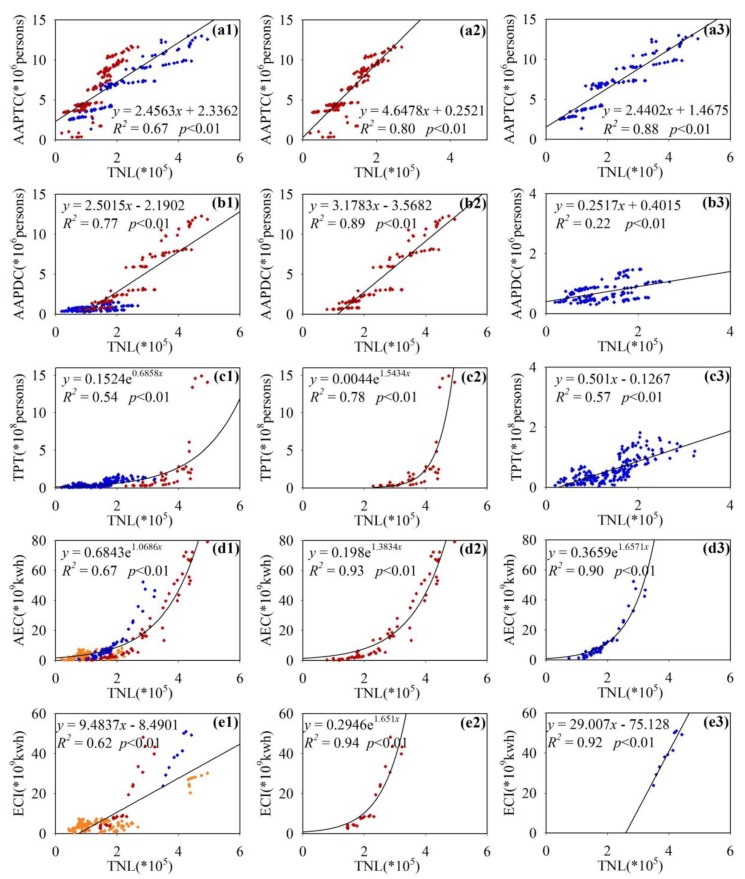
Regression analysis of socioeconomic parameters and TNL at different city levels. AAPTC and TNL for (**a1**) all cities; (**a2**) City group one (red points): inland cities; (**a3**) City group two (blue points): coastal economically developed cities. AAPDC and TNL for (**b1**) all cities; (**b2**) City group one: economically developed cities; (**b3**) City group two: economically underdeveloped cities. TPT and TNL for (**c1**) all cities; (**c2**) City group one: Beijing and Tianjin; (**c3**) City group two: other cities in Hebei Province. AEC and TNL for (**d1**) all cities; (**d2**) City group one: central core economic cities; (**d3**) City group two: industrial cities. ECI and TNL for (**e1**) all cities; (**e2**) City group one: industrial cities; (**e3**) Tianjin City.

Figure 6 has analysed the effects of eight socioeconomic parameters on artificial lighting. Driving factors had a certain correlation with artificial lighting in 13 cities. However, there were large differences in AAPTC (Figure 6a), AAPDC (Figure 6b), TPT (Figure 6e), AEC (Figure 6f) and ECI (Figure 6g) in different cities. It is difficult to reveal the influence of driving factors on the entire study area by using a single fitting model. Therefore, to explore the effects of different driving factors on artificial lighting in different city groups based on Figure 6, the study divided all 13 cities into different city groups according to their differences in economy, geographical location, and industrial structure. The fitting results are shown in Figure 7; the letter designations (a) to (e) of the Figure 7 subfigures correspond to (a)(b)(e)(f)(g) of Figure 6. 

The cities of AAPTC were divided into two groups. City group one includes Chengde, Zhangjiakou, Hengshui, Xingtai, Handan, Baoding, and Shijiazhuang, as shown in Figure 7(a2); City group two includes Beijing, Tianjin, Tangshan, Cangzhou, Langfang, and Qinhuangdao, as shown in Figure 7(a3). The *R*^2^ of city group one (0.80) and city group two (0.88) were both high, more than that of all cities (*R*^2^ = 0.67), which further illustrated that there were differences in the artificial lighting effects on AAPTC at different city levels. City group two mainly included the economic core areas of central and eastern regions and *R*^2^ was higher than that of city group one, with a relatively underdeveloped economy. Moreover, city group two includes coastal cities, such as Qinhuangdao, Tianjin, Tangshan, and Cangzhou, and city group one is mainly inland regions. For coastal cities, TNL grows faster with the growth of AAPTC, and the *R*^2^ is higher. The impact of AAPDC had large urban differences. As shown in Figure 7(b1), the blue points mainly represent economically underdeveloped cities, including Handan, Cangzhou, Zhangjiakou, Baoding, Xingtai, Qinhuangdao, Chengde, and Hengshui. The red points represent economically developed cities, including Beijing, Tianjin, Tangshan, Langfang, and Shijiazhuang. Figure 7(b2) shows that TNL was linearly related to AAPDC in economically developed cities, with strong correlation (*R*^2^ = 0.89), whereas the correlation of TNL and AAPDC was quite weak in economically underdeveloped cities (*R*^2^ = 0.22). Moreover, influenced by economically underdevelopment cities, the overall correlation was weakened (*R*^2^ = 0.77), weaker than that of city group one (*R*^2^ = 0.89).

Figure 7(c1) shows that TPT had large differences in different cities. For the two core economic cities of Beijing and Tianjin, TPT was significantly higher than for the other cities. Therefore, this study categorized Beijing and Tianjin as city group one and the remaining 11 cities in Hebei Province as city group two. Figure 7(c2) shows that TPT had an obvious exponential correlation with TNL in Beijing and Tianjin (*R*^2^ = 0.78), whereas the TPT in city group two was quite low and densely distributed with weak correlation. However, the overall TPT of city group two was small, and the impact on urban artificial lighting was relatively small (*R*^2^ = 0.57).

AEC includes all kinds of electricity consumption such as electricity for urban residents, commercial electricity, and industrial electricity. Therefore, AEC varies greatly among cities, and it is difficult to explore the impact of AEC on TNL with a single fitting model. Two types of city groups were distinguished based on the type of city and industrial structure. City group one represents central core economic cities, including Beijing, Tianjin, Langfang and Baoding, with high economic level and dense population. The region had high AEC and had a strong correlation with TNL (*R*^2^ = 0.93). City group two represents industrial cities, including Tangshan, Xingtai and Shijiazhuang, which had very large industrial electricity consumption. AEC in city group two was exponentially related to TNL, and the correlation was high (*R*^2^ = 0.90). The yellow points in Figure 7(d1) represent cities with low economic development and industrial level. In these cities, AEC was small and the correlation with TNL was quite weak.

Due to ECI being closely related to the urban industrial structure, there were large differences between various cities, as shown in Figure 7(e1). The cities were divided into different groups according to their industrial level. City group one represents typical industrial cities, including Tangshan, Shijiazhuang, and Xingtai. In these industrial-dominated cities, ECI had a quite strong correlation with TNL (*R*^2^ = 0.94). However, in another industrial city, Tianjin City, the impact of ECI on TNL was also strong (*R*^2^ = 0.92) and TNL in Tianjin was larger than that in city group one. The yellow points in Figure 7(e1) mainly represent cities with low industrial levels. Among them, some yellow points with high TNL and declining ECI represent Beijing.

**Figure 8:**
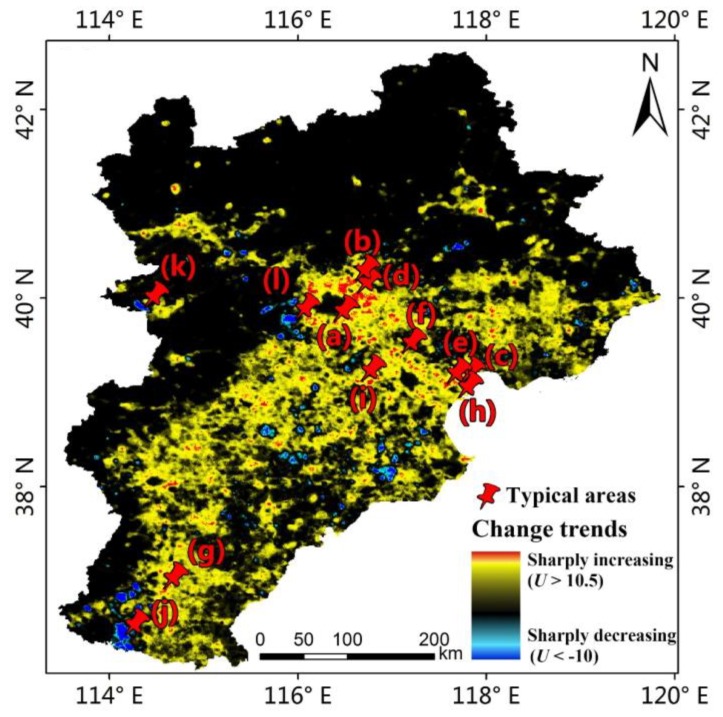
The locations of typical areas where nighttime light changes sharply.

The above analysis validated the correlation between artificial lighting and socioeconomic data such as population, economy, electricity consumption, and traffic. In the past 21 years, artificial lighting in some areas has sharply changed, as shown in Figure 3. To directly validate the driving factors of artificial lighting change in the Beijing-Tianjin-Hebei region, this study combined nighttime light data and high-resolution satellite images to monitor the land cover changes in the significant areas of nighttime light change. Figure 8 shows the locations of typical areas in which artificial lighting has sharply changed; areas (a) to (i) represent the sharply increasing areas, and areas (j) (k) (l) represent the sharply decreasing areas. 

**Figure 9:**
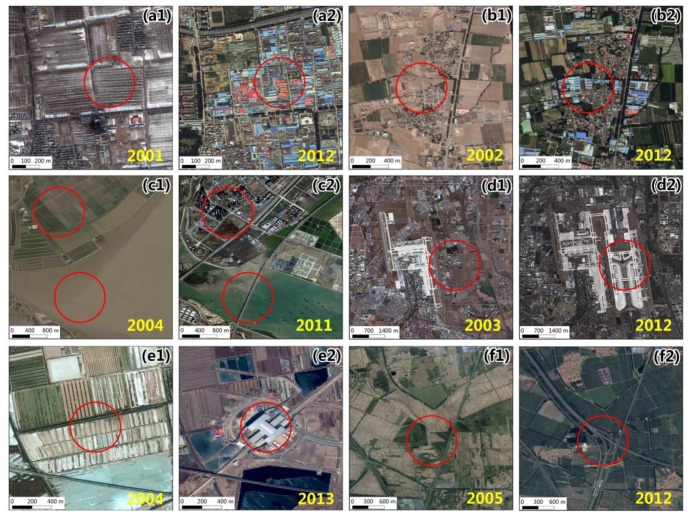

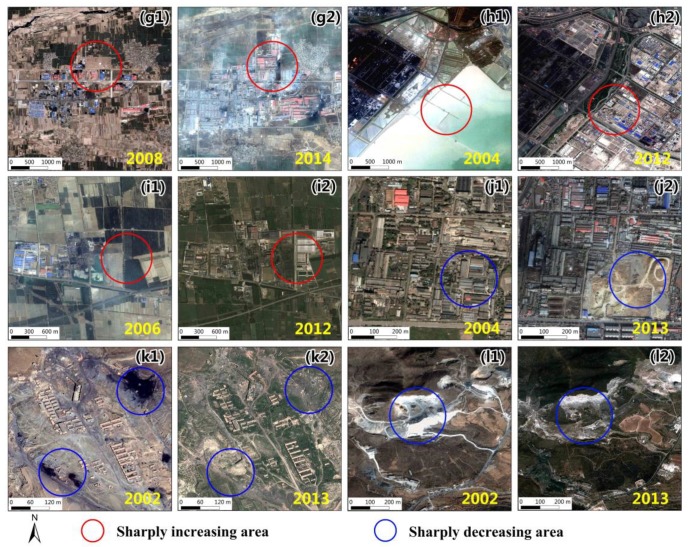
Validation of the typical areas where nighttime light has sharply changed. Sharply increasing areas: (**a1**–**c2**) urban expansion; (**d1**–**f2**) traffic development, including airports, railway stations and highways; (**g1**–**i2**) industrial growth. Sharply decreasing areas: (**j1, j2**) factory demolition; (**k1**–**l2**) shutdowns of coal mines.

The specific results of land cover changes are shown in Figure 9, where high-resolution satellite images for two dates are obtained from Google Earth. The letter designations of Figure 9 Subfigures correspond to those of Figure 8. Figure 9a–c represent the urban expansion, which brought about the growth of population and residential electricity consumption, a major cause of artificial lighting growth. Figure 9d–f represent the construction of transportation infrastructure, including airports, railway stations and highways. Large transportation hubs caused the growth of artificial lighting and also proved the impact of economy, population, and traffic on artificial lighting. Figure 9g–i show that the developments of steel mills and other industries brought about sharp growth in artificial lighting, and Figure 9j–l show that shutdowns of factories and coal mines resulted in the significant decrease of artificial lighting. All these results have proven the significant correlation between industries and nighttime light. 

## 5. Discussion

As one of the most developed regions in China, the Beijing-Tianjin-Hebei region has experienced unprecedented urbanization and economic growth [30]. However, the unreasonable use of artificial lighting facilities shows an explosive increasing trend and has gradually become a potential environmental problem [4,5,18]. Research on artificial lighting is of great significance for the prevention and treatment of light pollution issues. The change trend of nighttime lights has been reported in previous studies. Most of them have only explored the regional spatiotemporal variability of nighttime lights using time-series nighttime light imagery [4,18,20,22,39] or have analysed environmental pollution problems caused by nighttime lights [5,6,40]. A comprehensive study of the driving factors of nighttime lights with respect to socioeconomics is lacking. Many studies have explored the correlations between nighttime light and socioeconomical indices. However, previous studies have mainly employed nighttime light data to model socioeconomic phenomena, such as urban expansion [14], economic development [23], energy consumption [24], population density [25] and transportation mobility [26], and there has been a lack of studies that coupled nighttime light images and socioeconomic data to comprehensively explore the driving factors of artificial lighting changes. This study employed the driving factors according to different city groups distinguished by differences in urban economy, population density, geographical location, and industrial structure. High-resolution remote sensing images were used to explore reasons of significant artificial lighting change.

In the analysis of driving factors, this study not only selected different socioeconomic parameters according to the actual situation of the Beijing-Tianjin-Hebei region but also selected the parameters of different statistical units such as population parameters and economic parameters. The population parameters included AAPTC and AAPDC, for which the AAPTC had a large difference at different city levels. The correlation of economically developed cities and coastal cities was higher than that of economically underdeveloped cities and inland cities, which indicated that the impact of AAPTC on TNL was not only related to the urban economic level but also to geographical distribution [20]. The correlation of AAPDC was stronger in the entire study area, especially in economically developed cities. The results found that nighttime light strongly correlated with population density [32], and the AAPDC had larger population density in economically developed cities [41], whereas in economically underdeveloped cities, the correlation of AAPDC (*R*^2^ = 0.22) was weaker than that of AAPTC (*R*^2^ = 0.80). This may relate to the population distribution patterns of cities. In economically underdeveloped areas, the populations in surrounding rural and suburban areas are relatively high, which is an important source of nighttime lighting [4]. Differently than population parameters, economic parameters were exponentially related to TNL due to the accelerated development of China’s economic development during the study period. TPT had a relatively weaker impact on artificial lighting compared with other parameters in the entire study area; and the correlation varied with the city level, such as Beijing and Tianjin, which may have been caused by the “spatial spillover effect” [42]. The difference in electricity consumption was most significant, especially for ECI, and showed a weak correlation (*R*^2^ = 0.62). Industrial development in the Beijing-Tianjin-Hebei region was uneven [43]. In the main industrial cities, such as Tangshan, Shijiazhuang, Xingtai and Tianjin, the correlation was quite strong (*R*^2^ was above 0.90), indicating that the impact of industry on artificial lighting, as has been confirmed in previous studies [20,44]. Different from other cities, ECI had declined in Beijing with the growth of TNL, which may be related to the Olympic Games in 2008, with most of the factories in Beijing moved away. The study explored the impacts of a single driving factor on nighttime lights under different city groups of different statistical units; and the future research will focus on multi-source regression analysis [45] to investigate the combined effects of multiple driving factors. 

Although this study was conducted for the Beijing-Tianjin-Hebei region, it has broad applicability for research on the spatiotemporal variability of nighttime lights and the exploration of driving factors. The methods and framework can also be used for nighttime light monitoring and investigation in other regions of the world. New nighttime light sensors, such as the Visible Infrared Imaging Radiometer Sensor carried by the Suomi National Polar Orbiting Partnership (NPP/VIIRS) [46], the commercial EROS-B satellite [47], Jinlin 1-03 [48], and Luojia 1-01 [49,50,51], can contribute to the investigation of the characteristics and causes of nighttime light pollution at finer scales. In addition, NPP/VIIRS has good time continuity with DMSP/OLS. The combination of long-term DMSP/OLS images with the latest sensors to achieve normalization of long-time nighttime light remote sensing images is a further trend in artificial light investigations.

## 6. Conclusions

DMSP/OLS nighttime light images during 1992 to 2012 were selected and combined with the Mann-Kendall non-parametric test, nighttime light indices, standard deviation, socioeconomic data and high-resolution satellite images to investigate artificial lighting spatiotemporal variability and driving factors in Beijing-Tianjin-Hebei region. The main findings were as follows:

(1) In the Beijing-Tianjin-Hebei region, artificial lighting generally showed an increasing trend, which was most significant in the Beijing-Tianjin-Langfang triangular region. Artificial lighting had a decreasing trend in only a few areas, such as the west of Handan City, the southeast of Cangzhou City, and the suburbs of Beijing City and Zhangjiakou City.

(2) The changes of artificial lighting in the Beijing-Tianjin-Hebei region were relatively stable. Most of the study area had low fluctuations, and only a small part of the study area fluctuated greatly. The changes of artificial lighting in the northwest region were slow, whereas in the southeast region, the economic development was rapid and artificial lighting rapidly changed. Among them, Beijing, Tianjin and Tangshan had the highest fluctuations in artificial lighting and high growth rates.

(3) TNL, MNL, and SDNL of prefecture-level cities in the Beijing-Tianjin-Hebei region had an overall increasing trend. Beijing, Tianjin, and Tangshan had the largest TNL. Chengde and Zhangjiakou, the two economically underdeveloped cities, had the fastest growth rates of artificial lighting. The density of artificial lighting was related to the administrative area, such as TNL of Langfang City was low but MNL was relatively high. There was a large spatial heterogeneity of artificial lighting in Beijing, Tianjin, Langfang and other economically developed cities, and the spatial heterogeneity of artificial lighting in Chengde and Zhangjiakou was the lowest. 

(4) The impacts of different socioeconomic parameters on artificial lighting were analysed. In the entire area, AAPDC was the most important factor that affected artificial lighting in the Beijing-Tianjin-Hebei region, followed by GRP, AEC, and HECURR, and TPT, which had the least impact on artificial lighting. For the different city levels, the driving factors were mutative, related to differences in the economies, geographical location, and industrial structure of cities. According to high-resolution satellite imagery, urban expansion, transportation infrastructure construction, and industries were the major reasons for the significant change of nighttime light.

With the development of economy, the environmental risks brought about by artificial lighting pollution cannot be ignored. In the future, a variety of remote sensing data sources, combined with ground observation data and government statistical reports, will allow quantitative analysis of the effects of artificial lighting on human health, animals, plants, and ecosystems and will aid the development of accurate and effective artificial lighting measures. The rational use of artificial light sources contributes to energy conservation, emissions reduction, and sustainable development and can help to build a liveable and ecologically green society.

## Figures and Tables

**Table 1:** Parameters of high-resolution satellite imagery.

Typical Area	Sensor	Resolution (m)	Acquisition Time	Cloud Cover (%)
a1	IKONOS	0.8	01-28-2001	0.0
a2	Pléiades-1	0.5	10-13-2012	0.0
b1	QuickBird	0.6	04-24-2002	0.0
b2	WorldView-2	0.5	09-14-2012	4.0
c1	QuickBird	0.6	08-28-2004	0.0
c2	WorldView-2	0.5	11-23-2011	0.0
d1	QuickBird	0.6	11-16-2003	0.0
d2	GeoEye-1	0.5	12-31-2012	0.0
e1	QuickBird	0.6	05-04-2014	0.0
e2	KOMPSAT-2	1	03-03-2013	0.0
f1	QuickBird	0.6	06-30-2005	3.0
f2	Pléiades-1	0.5	08-22-2012	0.0
g1	QuickBird	0.6	04-24-2008	2.0
g2	WorldView-2	0.5	05-04-2014	1.0
h1	QuickBird	0.6	08-28-2014	0.0
h2	GeoEye-1	0.45	05-31-2012	1.0
i1	IKONOS	0.8	06-14-2006	0.0
i2	GeoEye-1	0.48	09-29-2012	0.0
j1	QuickBird	0.6	09-07-2004	0.0
j2	WorldView-2	0.5	12-11-2013	0.0
k1	QuickBird	0.6	05-22-2002	0.0
k2	GeoEye-1	0.49	08-19-2013	0.0
l1	QuickBird	0.6	03-24-2002	0.0
l2	WorldView-2	0.5	04-06-2013	0.0
